# Supplementary material for: Identification of photoperiod-induced specific miRNAs in the adrenal glands of Sunite sheep (Ovis aries)
Source: Front Vet Sci. 2022 Jul 22;9:888207. doi: 10.3389/fvets.2022.888207 (PMC9354845; doi:10.3389/fvets.2022.888207)
Supplement: Supplementary file 1 [file Data_Sheet_1.ZIP › Supplementary file/miRDeep2 file.zip!/miRDeep2 file/Know_Structure/SP42A3/oar-mir-22.pdf]

miRBase precursor : oar-mir-22
Total read count : 90686
oar-miR-22-3p read count : 85788
remaining reads : 4898

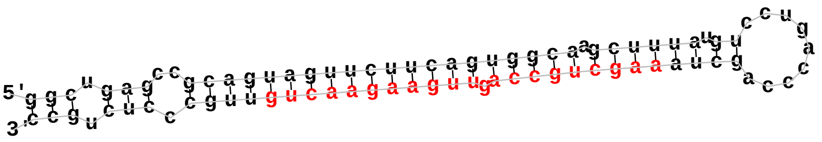

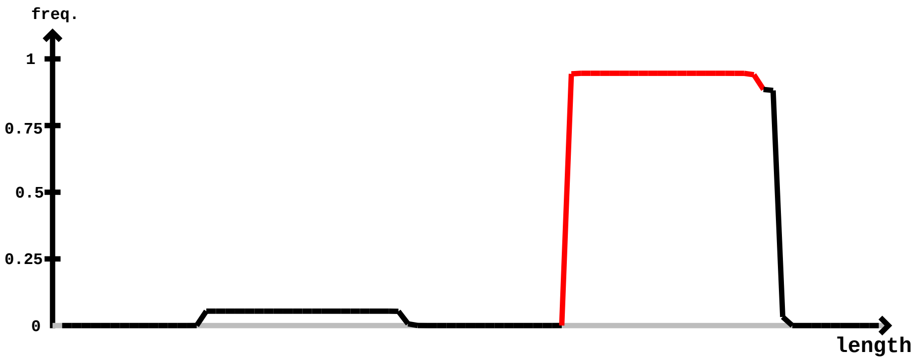

oar-miR-22-3p

```
5'- ggcugagccgcaguaguucuucaguggcaagcuuuauguccugacccagcuaaagcugccaguugaagaacuguugcccucugcc   -3'    exp
    (((.(((..((((((((((((((((((((.((((((.((.........))))))))))))).))))))))))))))).))).)))    reads     mm      sample
    .............uaguucuucaguggcaagcuu...................................................    2         0       seq
    .............uaguucuucaguggcaagcuuu..................................................    14        0       seq
    ..............aguucuucaguggcaagc.....................................................    1         0       seq
    ..............aguucuucaguggcaagcu....................................................    2         0       seq
    ..............aguucuucaguggcaagcuu...................................................    50        0       seq
    ..............aguucuucaguggcaagcuuu..................................................    4228      0       seq
    ..............aguucuucaguggcaagcuuua.................................................    567       0       seq
    ..............aguucuucaguggcaagcuuuau................................................    30        0       seq
    ...............guucuucaguggcaagcuuu..................................................    3         0       seq
    ................uucuucaguggcaagcuuuaugu..............................................    1         0       seq
    ...................................................aaagcugccaguugaaga................    1         0       seq
    ...................................................aaagcugccaguugaagaacu.............    7         0       seq
    ...................................................aaagcugccaguugaagaacugu...........    13        0       seq
    ....................................................aagcugccaguugaaga................    13        0       seq
    ....................................................aagcugccaguugaagaa...............    9         0       seq
    ....................................................aagcugccaguugaagaac..............    479       0       seq
    ....................................................aagcugccaguugaagaacu.............    4981      0       seq
    ....................................................aagcugccaguugaagaacug............    338       0       seq
    ....................................................aagcugccaguugaagaacugu...........    76845     0       seq
    ....................................................aagcugccaguugaagaacuguu..........    2917      0       seq
    .....................................................agcugccaguugaagaacu.............    8         0       seq
    .....................................................agcugccaguugaagaacug............    1         0       seq
    .....................................................agcugccaguugaagaacugu...........    153       0       seq
    .....................................................agcugccaguugaagaacuguu..........    21        0       seq
    ......................................................gcugccaguugaagaacugu...........    1         0       seq
    ......................................................gcugccaguugaagaacuguu..........    1         0       seq
```
